# Axillary lymph node dissection combined with radiotherapy for trichilemmal carcinoma with giant lymph node metastasis: A case report

**DOI:** 10.3389/fonc.2022.1019140

**Published:** 2022-12-08

**Authors:** Wenjie Lv, Dawen Zheng, Wenbin Guan, Ping Wu

**Affiliations:** ^1^ Department of Breast Surgery, Shanghai Jiao Tong University School of Medicine, Shanghai, China; ^2^ Department of General Surgery, Shanghai Jiao Tong University School of Medicine, Shanghai, China; ^3^ Department of Pathology, Shanghai Jiao Tong University School of Medicine, Shanghai, China

**Keywords:** trichilemmal carcinoma, axillary lymph node dissection, lymph node metastasis, radiotherapy, chemotherapy

## Abstract

**Background:**

Trichilemmal carcinoma (TC) is a rare malignancy with a poor outcome if local recurrence and distant metastasis occur. There is no treatment strategy for such a disease.

**Case presentation:**

We reported a complicated case of TC in the right lower abdomen with ipsilateral axillary and inguinal lymph node metastases. After surgery and radiotherapy, there has been no recurrence or metastasis in the follow-up to date.

**Conclusion:**

We believe that even though considered a tumor of low malignant potential, TC still has the risk of recurrence and metastasis, and the lymph node status should be identified if a high suspicion or diagnosis is made. Regional lymph node dissection followed by local radiotherapy is recommended as the optimal treatment strategy for patients with lymph node metastases of TC. Screening for metastasis and close follow-up are indispensable for improving prognosis.

## Introduction

Trichilemmal carcinoma (TC) is a rare malignant tumor of the hair follicles that presents as a papule, nodule, polyp, or ulcer, which needs to be differentiated from squamous cell carcinoma, basal cell carcinoma, malignant melanoma, keratoacanthoma, trichoblastomas, and lipomas. Local recurrence and distant metastasis are still possible despite slow clinical progression. There is currently no treatment strategy (1). In this paper, we report a complicated case of TC in the right lower abdomen with ipsilateral axillary and inguinal lymph node metastases; the former grew to 7 cm in diameter. After surgery and radiotherapy, there has been no recurrence or metastasis in the follow-up to date. Our paper aims to provide references for the treatment of TC and its lymph node metastasis.

## Clinical data

**Figure 1:**
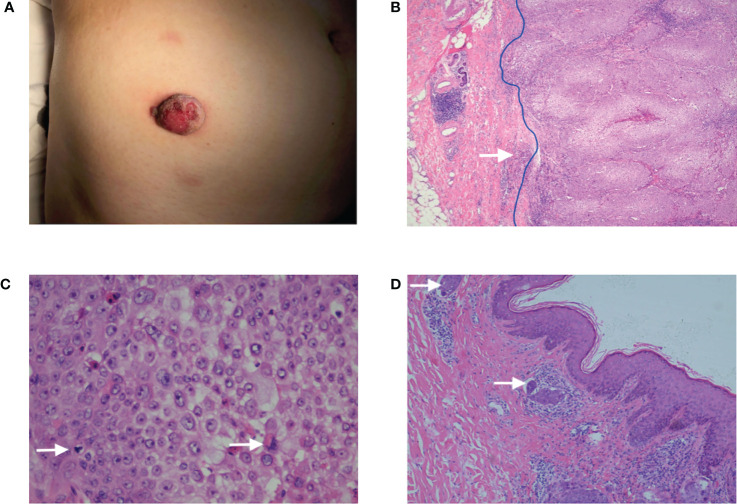
**(A)** A reddish plaque on the skin of the right lower abdomen with a diameter of 2.5 cm. **(B)** The HE ×40: The tumor presented as an exo-endophytic proliferation with a dermal multinodular, well-circumscribed growth connected to the epidermis (blue line), and an invasive focus of frankly atypical epithelium could be observed (white arrow). **(C)** The HE ×400: The tumor was composed of clear, monomorphic cells with mitosis. **(D)** The HE ×100: Tumor thrombus could be observed (white arrow).

**Figure 2:**
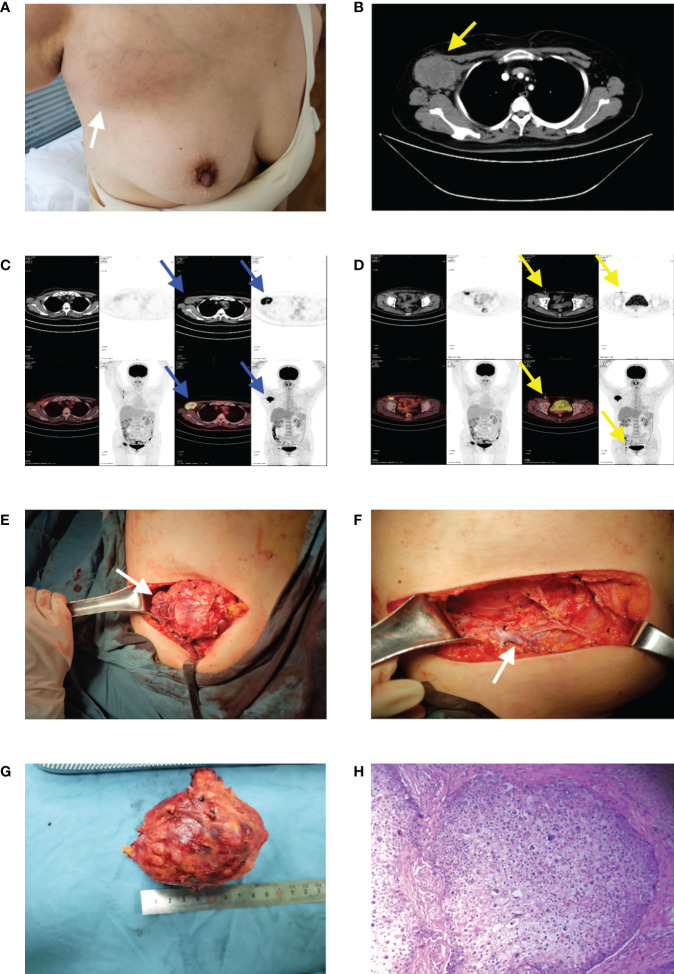
**(A)** A mass in the right axilla. **(B)** Preoperative enhanced CT revealed a soft tissue density mass (yellow arrow) in the right axilla with a size of 6.9 × 5.2 cm. **(C, D)** PET-CT showed the mass as axillary lymph node metastasis (blue arrows) and another metastasis located at the ipsilateral inguinal lymph node (yellow arrows) which were characterized by shapes of nuclide accumulations in PET-CT image. **(E)** An intraoperative view from above, showing the tumoral invasion of the pectoralis major and minor muscles (white arrow). **(F)** An intraoperative view from above, showing the axillary vein (white arrow) after ALND. **(G)** The tumor was 7 cm in diameter. **(H)** The HE ×100 showed the tumor was characterized by a proliferation of tumoral lobules composed of large, atypical cells with clear cytoplasm.

**Figure 3:**
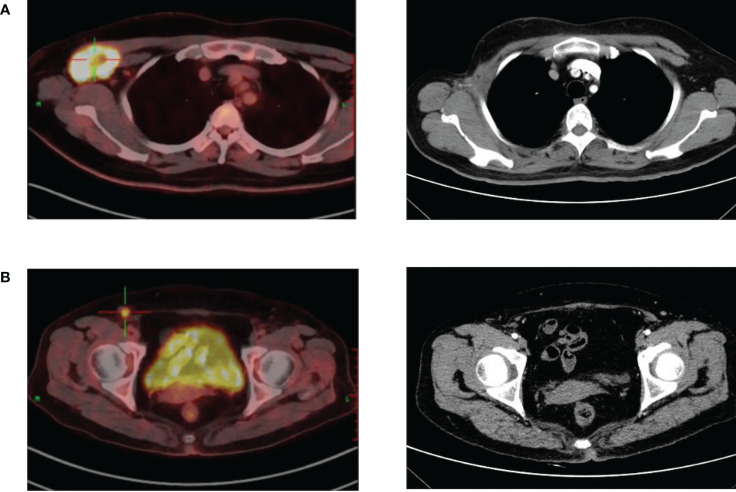
**(A)** An enhanced CT of the chest showed no recurrent manifestation in the right axilla compared with preoperative PET-CT. **(B)** CT follow-up revealed the disappearance of the lesion in the right inguinal region after adjuvant radiotherapy compared with preoperative PET-CT.

A 63-year-old female found a peanut-sized red plaque occasionally on her right lower abdomen skin in February 2019, and after 5 months, the plaque suddenly increased to 2.5 cm with purulent discharge exudation and bled after rubbing, but with no pain (**Figure 1A**). No family history of the patients was recorded. In August 2019, the patient underwent local resection with an excision margin >2 cm from the tumor. The postoperative pathological diagnosis was trichilemmal carcinoma with margin-free but with tumor thrombus **(** **Figures 1B-D** **)**. In March 2020, the patient found a mass in the right axilla, confirmed as axillary lymph node metastasis of TC by biopsy pathology. PET-CT showed another metastasis located at the ipsilateral inguinal lymph node. The right axillary mass was progressively increasing to 6.9 × 5.2 cm after two courses (21 days per course) of chemotherapy (docetaxel + DDP) (**Figures 2A-D**). Right axillary tumor resection, axillary lymph node dissection (ALND), and right inguinal lymph node biopsy were performed in June 2020. The tumor was 7 cm in diameter, invading the pectoralis major and minor muscles and the axillary vein’s sidewall (**Figures 2E-G**), with multiple enlarged lymph nodes in the axilla. The postoperative pathology confirmed the tumor and axillary lymph nodes as metastases (12/28 positive) (**Figure 2H**). The inguinal lymph nodes (1/2 positive) were also confirmed as having metastasized from TC after resection. We performed genetic studies of lymph node metastases, in which PTEN (37.60% rate of mutations, the percentage of mutated genes in all genes detected) and TP53 (58.10% rate of mutations) mutations were found but no germline mutations were detected. Postoperative adjuvant radiotherapy and oral capecitabine chemotherapy for 5 weeks were prescribed with the consideration that oral capecitabine combined with radiation might produce better outcomes for local-regional control (2). The right axillary, supraclavicular, and inguinal regions received a dose of 180 Gy in 25 fractions. The boost dose for the right axillary and supraclavicular regions was 230 Gy in 25 fractions. All dose schedules were given 5 days per week. An enhanced CT of the chest in August 2021 showed no recurrent manifestation in the right axilla (**Figure 3A**); however, right inguinal lymph nodes were confirmed to have metastasis of TC in regular follow-ups. Adjuvant radiotherapy for the right inguinal region with a dose of 180 Gy in 25 fractions was administered for 5 weeks. The boost dose for the tumor bed was 220 Gy in 25 fractions. All dose schedules were given 5 days per week. The patient tolerated the treatment well, and no severe adverse events occurred except for inflamed skin in the right axilla, which recovered soon at the end of radiation. After radiotherapy, regular ultrasound and CT follow-up revealed the disappearance of the lesion in the right inguinal region **(** **Figure 3B** **)**. The patient has shown no signs of recurrence or metastasis for one year since last radiotherapy.

## Discussion

Trichilemmal carcinoma, derived from the outer root sheath epithelium of the hair follicle, occurs mostly on the head, face, and neck, rarely on the extremities or trunk. It is more common in older people, and a slight majority of cases were reported in men (3). The pathogenesis is not completely understood to date. TP53 mutations were reported to be identified in patients with an aggressive clinical course that suggested similar pathogenesis in TC with other skin cancers (4). Many risk factors, including UV and ionizing radiation, previous trauma or scarring, genetic disorders, and immunosuppression for solid organ transplantation, have been identified (3).

In our case, the tumor presented as an exo-endophytic proliferation with a dermal multinodular, well-circumscribed growth connected to the epidermis and an invasive focus of frankly atypical epithelium. Especially it needs to be differentiated from squamous carcinoma and porocarcinoma. Squamous carcinoma usually shows an invasive growth, not a dermal multinodular, well-circumscribed growth. Porocarcinomas can appear as dermal multinodular, well-circumscribed growths with intraepithelial nodules. But predominantly clear cells are quite rare. Some cases can show duct structure.

Complete surgical excision with tumor-free margins is the most common treatment, and re-excision is strongly recommended if the histologic excision margins are not totally free (5, 6). Mohs micrographic surgery (MMS) has been shown to be an ideal option recently because it provides a tissue-sparing method for complete surgical removal of the tumor while preserving the surrounding healthy tissue (7–9).

TC is usually considered to have an indolent course and benign clinical evolution. Regional and distant metastases of TC are rarely reported. The most frequent metastatic organ is the regional lymph nodes, which are associated with poor prognosis (10, 11). In our case, metastases to the ipsilateral axillary and inguinal lymph nodes were identified 7 months after the lesion’s resection. Therefore, we strongly recommend evaluating the ipsilateral superficial lymph nodes under any diagnostic suspicion of TC, as well as the use of PET-CT if necessary. If a high risk of recurrence in postoperative pathology presents (such as intravascular tumor thrombus), close follow-up of the ipsilateral lymph nodes is required.

A survival study of TC (12) reported systemic chemotherapy should be considered when distant tumor metastasis is confirmed, potentially useful for monitoring disease progression. When ipsilateral axillary and inguinal lymph node metastases in our patient were confirmed, adjuvant chemotherapy with the TP regimen was performed. However, the axillary mass increased from 5.5 cm to 7 cm after chemotherapy. Oral capecitabine chemotherapy after ALND also failed to control the progression of inguinal lymph node metastasis, suggesting chemotherapy had limited efficacy in treating distant metastasis.

Surgical treatment for lymph node metastases of TC is rarely reported. We believe that regional lymph node dissection can also greatly improve the prognosis, and surgical intervention is highly recommended even if lymph node metastasis occurs. A previous study (13) suggested initial surgical excision followed by adjuvant radiation offered excellent local-regional control for patients with skin carcinomas, especially those with high-risk features including lymph node metastasis, positive margins, high grade, multi-focal disease, and recurrent disease. In our case, the patient underwent local radiotherapy after ALND and inguinal lymph node dissection. The recurrent lesions in the right groin area disappeared after radiotherapy, and there has been no evidence of metastasis or recurrence in the follow-up so far. This case indicated that regional lymph node dissection combined with local radiotherapy could achieve an ideal prognosis in patients with lymph node metastases of TC. For cases with local recurrence or difficult surgery, radiotherapy could be considered as a clinical option instead of reoperation to avoid surgical risks and complications.

Herein, we report an extremely rare case of extensive lymph node metastases after surgery for TC, with a favorable outcome achieved by regional lymph node dissection followed by radiotherapy. We believe that even when considered to have low malignant potential, TC still has the risk of recurrences and metastases, and the lymph node status should be determined if a high suspicion or diagnosis is made. Regional lymph node dissection followed by local radiotherapy is recommended as the optimal treatment strategy for patients with lymph node metastases of TC. Screening for metastasis and close follow-up are indispensable for improving prognosis.

## Data availability statement

The raw data supporting the conclusions of this article will be made available by the authors without undue reservation.

## Ethics statement

Written informed consent was obtained from the individual for the publication of any potentially identifiable images or data included in this article.

## Author contributions

WL, DZ, and PW collected the clinical data. WL and DZ reviewed the MR and CT images. WG reviewed the pathology findings. DZ drafted the manuscript. WL and PW revised the manuscript. All authors read and approved the final manuscript.

## References

[1] Evrenos MK Kerem H Temiz P Ermertcan AT Yoleri L . Malignant tumor of outer root sheath epithelium, trichilemmal carcinoma. clinical presentations, treatments and outcomes. Saudi Med J (2018) 39(2):213–6. doi: 10.15537/smj.2018.2.21085 PMC5885101 29436573

[2] Allegra CJ Yothers G O'Connell MJ Beart RW Wozniak TF Pitot HC . Neoadjuvant 5-FU or capecitabine plus radiation with or without oxaliplatin in rectal cancer patients: A phase III randomized clinical trial. J Natl Cancer Inst (2015) 107(11):djv248. doi: 10.1093/jnci/djv248 26374429 PMC4849360

[3] Hamman MS Brian Jiang SI . Management of trichilemmal carcinoma: an update and comprehensive review of the literature. Dermatol Surg (2014) 40(7):711–7. doi: 10.1111/dsu.0000000000000002 25111341

[4] Ha JH Lee C Lee KS Pak CS Sun CH Koh Y . The molecular pathogenesis of trichilemmal carcinoma. BMC Cancer (2020) 20(1):516. doi: 10.1186/s12885-020-07009-7 32493317 PMC7271408

[5] Jia Q Yuan Y Mao D Wen G Chen X . Trichilemmal carcinoma of the scalp in a young female: A case report. Clin Cosmet Investig Dermatol (2022) 15:139–43. doi: 10.2147/CCID.S349797 PMC8805741 35115802

[6] Kanitakis J Euvrard S Sebbag L Claudy A . Trichilemmal carcinoma of the skin mimicking a keloid in a heart transplant recipient. J Heart Lung Transplant (2007) 26(6):649–51. doi: 10.1016/j.healun.2007.03.001 17543793

[7] Rodríguez-Jiménez P Jimenez YD Reolid A Sanmartın-Jimenez O Garces JR Rodríguez-Prieto MA . State of the art of mohs surgery for rare cutaneous tumors in the Spanish registry of mohs surgery (REGESMOHS. Int J Dermatol (2020) 59(3):321–5. doi: 10.1111/ijd.14732 31777957

[8] Tolkachjov SN . Adnexal carcinomas treated with mohs micrographic surgery: A comprehensive review. Dermatol Surg (2017) 43(10):1199–207. doi: 10.1097/DSS.0000000000001167 28445202

[9] Tolkachjov SN Hocker TL Camilleri MJ Baum CL . Mohs micrographic surgery in the treatment of trichilemmal carcinoma: the Mayo clinic experience. J Am Acad Dermatol (2015) 72(1):195–6. doi: 10.1016/j.jaad.2014.10.007 25497926

[10] Maya-Rico AM Jaramillo-Pulgarin C Londono-Garcia A Pena-Zuniga B . Locally aggressive trichilemmal carcinoma. Bras Dermatol (2018) 93(4):579–81. doi: 10.1590/abd1806-4841.20187461 PMC6063117 30066770

[11] Xie Y Wang L Wang T . Huge trichilemmal carcinoma with metastasis presenting with two distinct histological morphologies: A case report. Front Oncol (2021) 11:681197. doi: 10.3389/fonc.2021.681197 34552863 PMC8452038

[12] Zhuang SM Zhang GH Chen WK Chen SW Wang LP Li H . Survival study and clinicopathological evaluation of trichilemmal carcinoma. Mol Clin Oncol (2013) 1(3):499–502. doi: 10.3892/mco.2013.74 24649199 PMC3915649

[13] Wang LS Handorf EA Wu H Liu JC Perlis CS Galloway TJ . Surgery and adjuvant radiation for high-risk skin adnexal carcinoma of the head and neck. Am J Clin Oncol (2017) 40(4):429–32. doi: 10.1097/COC.0000000000000178 PMC4504824 25599317

